# Biomarkers of Bladder Cancer: Cell-Free DNA, Epigenetic Modifications and Non-Coding RNAs

**DOI:** 10.3390/ijms232113206

**Published:** 2022-10-30

**Authors:** Stefan Harsanyi, Zuzana Varchulova Novakova, Katarina Bevizova, Lubos Danisovic, Stanislav Ziaran

**Affiliations:** 1 Institute of Medical Biology, Genetics and Clinical Genetics, Faculty of Medicine, Comenius University in Bratislava, Sasinkova 4, 811 08 Bratislava, Slovakia; 2 Institute of Anatomy, Faculty of Medicine, Comenius University in Bratislava, Sasinkova 2, 811 08 Bratislava, Slovakia; 3 Department of Urology, Faculty of Medicine, Comenius University in Bratislava, Limbova 5, 833 05 Bratislava, Slovakia

**Keywords:** bladder cancer, biomarkers, cell-free DNA, DNA methylation, non-coding RNA

## Abstract

Bladder cancer (BC) is the 10th most frequent cancer in the world. The initial diagnosis and surveillance of BC require a combination of invasive and non-invasive methods, which are costly and suffer from several limitations. Cystoscopy with urine cytology and histological examination presents the standard diagnostic approach. Various biomarkers (e.g., proteins, genes, and RNAs) have been extensively studied in relation to BC. However, the new trend of liquid biopsy slowly proves to be almost equally effective. Cell-free DNA, non-coding RNA, and other subcellular structures are now being tested for the best predictive and diagnostic value. In this review, we focused on published gene mutations, especially in DNA fragments, but also epigenetic modifications, and non-coding RNA (ncRNA) molecules acquired by liquid biopsy. We performed an online search in PubMed/Medline, Scopus, and Web of Science databases using the terms “bladder cancer”, in combination with “markers” or “biomarkers” published until August 2022. If applicable, we set the sensitivity and specificity threshold to 80%. In the era of precision medicine, the development of complex laboratory techniques fuels the search and development of more sensitive and specific biomarkers for diagnosis, follow-up, and screening of BC. Future efforts will be focused on the validation of their sensitivity, specificity, predictive value, and their utility in everyday clinical practice.

## 1. Introduction

Bladder cancer (BC) is the 10th most common cancer worldwide, with over 573,000 new cases in 2020 [1]. Cellular origin in the urothelium accounts for more than 90% of all BCs [2]. These lesions range from benign, through small tumors, to aggressive and malignant neoplasms with lymphatic or vascular invasion, high recurrence rates with poor response to treatment, and bad prognosis [3,4,5].

The worldwide estimation of BC incidence for 2020 by The International Agency for Research on Cancer (IARC) reported that in males, BC is the seventh most frequent malignancy, while in females the frequency drops over three to four-fold, depending on the region. In the European Union, the age-standardized incidence rate is 20 for men and 4.6 for women as opposed to estimates from the year 2018, where the age-standardized incidence stood at 30.9 and 6.5, respectively [6,7,8]. According to the American Cancer Society (ACS), in the USA, approximately 90% of BC cases are patients older than 55 years and the average age at the time of diagnosis is 73 years. Additionally, BC is rated as the fourth most common malignancy in men, while the risk for women is 3.3-fold lower. The estimates for BC incidence in 2021 in the USA are 83.730 new cases (men—64.280, women—19.450) and 17.200 deaths (men—12.260, women—4.940), which points to a slight reduction in new cases in both men and women [9]. This phenomenon has also been observed in other countries [10,11,12]. However, discrepancies in worldwide reports of incidence and mortality are partially caused by different methodologies in data collection and analysis.

Tissue for histological or immunohistochemical examination is obtained by transurethral resection (TUR) of the papillary lesion or by multiple biopsies in the carcinoma in situ (Tis) stage. Later, the tumor tissue of individual patients is evaluated using the EORTC (European Organization for Research and Treatment of Cancer) scoring system for possible progression and recurrence [13,14]. According to the guidelines by the European Association of Urology (EAU), at the time of diagnosis, about 25% of patients present with muscle-invasive bladder cancer (MIBC), while the majority, about 75% of patients present with non-muscle invasive bladder cancer (NMIBC) [15,16]. These lesions are located in the mucosa (Ta, Tis) or submucosa (T1). The frequency of NMIBC is higher in younger patients (under 40) [17]. According to ACS, in the USA, approximately 50% of patients present with non-invasive or in situ cancers [18]. NMIBC has a one-to-five-year recurrence rate of 15–61% and an up to 17% chance of progression to MIBC within the first five years [19,20]. Additionally, even with combination treatment, the prognosis of patients remains unclear.

Etiopathogenesis of BC is not thoroughly explained, but many researchers consider this pathology similarly affected by environmental, mechanical, and genetic factors or rather, predisposition. Since the most common initial complaints of patients are macroscopic hematuria, dysuria, and symptoms similar, but unrelated to urinary tract infections (UTIs), researchers concluded that certain risk factors are in a causative relationship with BC [21]. These risk factors associated with BC include tobacco smoking, dietary factors, work-related exposure to chemicals (irritants, teratogens, mutagens), secondary cancer post-radiotherapy, chronic or frequent UTIs, or bladder schistosomiasis, and finally—race, gender, and genetic factors [22,23,24,25].

Diagnosing patients with NMIBC in the EU requires a combination of invasive (cystoscopy) and non-invasive methods (upper urinary tract imaging, and urine cytology). However, the management of patients from initial symptoms presentation to final diagnosis confirmed by tumor histology is a long process, and its speed and quality can positively or negatively affect patient prognosis. Patients undergoing regular preventive examinations are questioned on personal and family history, have their urine tested, undergo urinary cytology, and scheduled cystoscopies [26,27]. Urinary cytology, even if user-dependent is more sensitive for high-grade (HG) tumors and works best in conjunction with cystoscopy. On the other hand, patients not regularly examined tend to visit the physician with visible, often painless hematuria as their primary symptom. Irritative voiding and lower UTI symptoms are present to a lesser extent, especially present in the Tis stage. Imaging methods such as CT urography and ultrasonography (USG) are used to detect abnormalities in the urinary tract [28,29]. Magnetic resonance imaging (MRI) is also gaining interest in BC diagnostic process [30,31,32].

Due to the aforementioned limitations in the diagnosis, treatment, and management of BC patients, there is an urgent, long-standing need for better, more reliable, more precise examination methods that could provide a better understanding of individual cancer types, not only in the case of BC. Information about recurrence, progression, and prognosis is vital for the reliable stratification of patients and the future management of cancer as a whole. In this search for better predictive factors, various types of biomarkers are being studied for their association with BC [33,34]. Although, according to the EUA complex approaches such as the stratification of patients based on molecular classification, are promising but are not yet suitable for routine application [26,35].

In this review, we focused on published gene mutations, especially in DNA fragments, but also epigenetic modifications, and non-coding RNA (ncRNA) molecules acquired by liquid biopsy. Apart from various biochemical markers contained in bodily fluids, extracellular vesicles (EVs) attract high interest, be it exosomes (30–150 nm) or microvesicles (100 nm–10 μm) [36]. These vesicles contain nucleic acids, proteins, lipids, and metabolites or other biomolecules [37,38]. Since EVs are plasmatic membrane structures, they can be internalized into cells [39]. One of their most important roles is the transportation of various RNA molecules and proteins, accompanied by different fragments of nucleic acids and due to this, they are being studied in the process of carcinogenesis [40,41]. The possibility to isolate EVs from bodily fluids gave way to examining ncRNAs, DNA fragments, and more [42]. As liquid biopsy poses the least invasive method, collection of exosome contents is preferred this way. In further sections, we will address biomarkers, that come from EVs acquired by liquid biopsy.

We performed an online search in PubMed/Medline, Scopus, and Web of Science using the terms “bladder cancer”, in combination with DNA, RNA, and epigenetic “markers” or “biomarkers” published until August 2022. If applicable, we set our sensitivity (SN) and specificity (SP) threshold to be at least 80%. Panel markers are specified as possible synergic diagnostic biomarkers, that could enhance each other’s potential in a diagnostic panel. Complementing SN/SP scores was the area under curve (AUC) score, which was equally set to 0.80 and more, to ensure high diagnostic potential. As genes and various RNA molecules are usually not evaluated by SN/SP or AUC, the threshold of inclusion has been set on a repeated statistical significance reported by researchers. In this review, we did not focus on commercially approved markers, as they are well-studied and often do not fit the required SN/SP score, save for the latest Uromonitor-V2 [43]. For illustration EAU-approved and FDA-approved markers and their pooled SN/SP or AUC scores are listed in Table 1 [44,45].

## 2. Cell-Free DNA

Cell-free DNA (cfDNA) obtained from the liquid biopsy is fragmented DNA originating from deteriorating cancer cells [53]. Evaluation of alterations in DNA structure is gaining interest in the identification of cancer heterogeneity and patient prognosis. The method of real-time PCR was used to analyze quantitative changes in gene products [54]. However, nowadays the most frequent method for detecting alteration in cfDNA utilizes whole-genome sequencing, including digital PCR and next-generation sequencing (NGS) [55]. Sangster et al. proposed a theory of mutually exclusive gene mutations, where one gene contains a mutation, while the other does not, similarly in reverse [56]. This way they located two mutually exclusive pairs: KDM6A and KMT2D and also KDM6A and RB1.

The most common alteration is DNA mutation in genes associated with the processes of embryogenesis, proliferation, cell cycle regulation, and apoptosis. Genes with a substantial amount of research in association with BC are presented in Table 2.

The fibroblast growth factor receptor 3 gene (FGFR3) encodes for protein containing an extracellular domain with either 2 or 3 immunoglobulin (Ig)-like domains, a transmembrane domain, and a cytoplasmic tyrosine kinase domain that interacts with fibroblast growth factor and plays an important role in many important cellular processes, including regulation of proliferation, differentiation, apoptosis, angiogenesis), wound healing, and embryogenesis [63]. It was demonstrated that ectopic activation of FGFR3 is associated with several cancers, including multiple myeloma, cervical cancer, and BC [64,65,66]. In the case of BC, somatic mutations and gene overexpression are prevalent and may hold value as prognostic markers and as a tool for patient selection. Alterations affecting FGFR3 signaling were detected more frequently in the urinary bladder than in any other cancer type. Several forms of activation were described. The most common mechanism is missense point mutation which shows a strong relation to low-grade (LG) and low-stage cancer [67,68]. It was also shown that mutant FGFR3 affects the cell cycle regulation and led to changes in cell junctions and cell adherence to proteins occurring in the urothelial basement membrane and adjacent connective tissue, and induces alteration in expression of the extracellular matrix modulators, all functions predicted to provide a selective advantage to cells in the process of initial stages of cancer development [69]. Another form of FGFR3 activation is the formation of fusion proteins that can contribute to genomic instability [70]. However, the frequency of FGFR3-TACC3 fusion genes and their prognostic role are still unknown. FGFR3 overexpression seems to be a more suitable prognostic marker. Several studies demonstrated that stage Ta and T1 BC show overexpression of FGFR3 in 70–80% of Ta and 40–70% of T1 tumors [71,72,73]. More recently it has been shown that FGFR3 overexpression was also associated with reduced response to Bacillus Calmette-Guerin treatment and the expression of FGFR3 correlated with NMIBC stage, with more frequent overexpression in pTa tumors [74]. A recent study utilizing a 6-gene panel comprising 3 mutations (FGFR3, TERT, and HRAS) and 3 methylation analyses (OTX1, ONECUT2, and TWIST1) resulted in significant findings in the case of SN/SP/AUC as seen in Table 2, however, HRAS mutation did not prove significant [57].

PVRL4 gene (Nectin-4) encodes for a 510-amino acid protein (Nectin cell adhesion molecule) which contains 1 predicted transmembrane domain, followed by a 139-residue cytoplasmic sequence. It is expressed in the placenta, trachea, and human skin [75]. Recently it is considered a new prognostic biomarker for several types of cancer. Anti-nectin-4 antibody–drug-conjugate has great potential as a therapeutic agent for metastatic urothelial carcinoma [76]. It was also demonstrated that PVRL4 was also strongly expressed in NMIBC suggesting the assessment of anti-nectin-4 as a promising biomarker during the initial stages of the disease. In another experimental study, overexpression of PVRL4 in luminal BC cell lines was found, which correlated with the expression of GATA3 [58]. Current advances in monoclonal antibody treatment allowed for an anti-nectin-4 antibody (Enfortumab vedotin) to be approved for BC therapy, as moderate to strong overexpression of PVRL4 has been observed in 60% of BC patients [77].

Cyclin E gene (CCNE1) encodes for protein which has a pivotal role in cell cycle progression and differentiation [78]. Moreover, Keyomarsi et al. showed that breast cancers, and some other solid tumors, display significant quantitative and qualitative alterations in cyclin E protein production [79]. Rothman et al. demonstrated overexpression of CCNE1 in HG bladder tumors. There is also preliminary evidence that CCNE1 amplification is associated with frequent TP53 mutation and aggressive clinical outcomes [80]. A recent study reported a positive correlation between disease progression and copy-number variations (CNV) in CCNE1 combined with CDKN2A deletion [59].

Cyclin-dependent kinase inhibitor 2A (CDKN2A) encodes proteins that regulate two important cell cycle regulators—p53 and RB1. It produces a cyclin-dependent kinase inhibitor p16(INK4), and p14(ARF), which is essential for binding the p53-stabilizing protein MDM2 [81]. Several studies provided evidence that CDKN2A mutations are associated with cancer development. For instance, Chan et al. showed that pathogenic germline variants in CDKN2A significantly increase the risk of cutaneous melanoma and these alterations are also occasionally associated with a rare melanoma-astrocytoma syndrome and may lead to the development of malignant melanomas and neural system tumors, such as astrocytomas and meningiomas [82]. A large deletion in CDKN2A has been reported in neurofibromas, giant cell tumors of bone, and multiple primary cancers including sarcomas [83,84]. Loss of heterozygosity (LOH) in the 9p region belongs to typical processes in the initial stages of bladder cancer development [85]. Moreover, LOH of CDKN2A in combination with down-regulation of the p16 is in good correlation with progression in NMIBC [86]. CDKN2A homozygous deletion is also associated with invasiveness in FGFR3-mutated urothelial bladder carcinoma [87]. In a recent study, Verma et al. demonstrated that CDKN2A in combination with CTSV and FOXM1 has great potential to be a promising predictive marker of BC progression with 95.5% sensitivity and 100% specificity [60].

The telomerase reverse transcriptase gene (TERT) encodes a protein that acts as a safeguard of genomic integrity and is responsible for telomere maintenance. In cancer, telomerase activity is elevated and as a result, there is no induction of telomere shortening which enables cells to overcome replicative senescence and escape apoptosis, which belongs to crucial steps in cancer development [88]. TERT gene mutations are typical for various cancers, including melanoma, acute myeloid leukemia, and BC [89,90,91]. It was shown that mutations in the TERT promoter region are the most common somatic lesions in BC and may affect patient survival and disease recurrence through modification by a common polymorphism [92]. Carrasco et al. assessed the increased presence of TERT mutations as a potential biomarker of cancer aggressivity and progression [93]. Furthermore, Descostes et al. showed that TERT mutations in urine samples might be helpful for the early detection of recurrence in BC, especially in NMIBC. Overall sensitivity was 80.5% and specificity 89.8% [61].

Pleckstrin homology domain-containing S1 gene (PLEKHS1) encodes a protein with a function that still remains unclear. However, there are indications that PLEKHS1 may be after TERT function and is involved in cancer development. Interaction with the insulin-like growth factor (IGF) axis has been suggested, as PLEKHS1 was associated with mild blood glucose elevation, insulin resistance, and obesity [94]. Pignot et al. designed a two-phase study, where tissue from 154 and 181 bladder tumors was tested for PLEKHS1 mutation and its mRNA overexpression. Mutations occurred in 25.0% and 33.0% of NMIBC, while for MIBC it was 32.2% and 37.8%, respectively [95]. Dudley et al. discovered PLEKHS1 mutation in 46% of tested BC subjects [62]. Mutations of PLEKHS1 along with TERT promoter and GPR126 intron 6 have been found elevated in BC patients [96].

## 3. Epigenetic Modifications

Epigenetic modifications include histone modification, DNA methylation, and effects of ncRNA. Histone modification is not thoroughly studied, but DNA methylation is an important epigenetic modification, which plays an important role in regulating gene expression at the transcriptional level [97]. In tumor research, it has been found that the change in DNA methylation leads to the abnormality of gene structure and function, which can provide an early warning for tumorigenesis. The list of epigenetically modified genes is listed in Table 3.

An improved urine DNA methylation panel of three biomarkers (PCDH17, POU4F2, and PENK) showed SN of 97% and SP of 87% in BC detection [98]. A study by El Azzouzi et al., showed a significant association between TWIST1 hypermethylation and BC recurrence and progression (66.67% and 80%), while hypermethylation of hTERT was found in 83.34% of recurrent and 80% of progression cases [99]. TWIST1 hypermethylation combined with SALL3 and CFTR showed an AUC of 0.86 and SN/SP of 90%/40% if combined with cytology [100]. DMRTA2 was reported as a new DNA methylation marker to detect BC [101]. Collected by urine-based liquid biopsies, this marker proved the most sensitive for the T1 and T2 stages. OTX1 along with TWIST1 and ONECUT2 examined by SNaPshot™ methylation analysis exhibited SN/SP of 97%/83% and AUC 0.92 for BC prediction in hematuria patients [102]. OTX1 methylation assay combined with FGFR3 and TERT mutation analysis yielded an SN of 57% for LG-NMIBC recurrence and 83% for pT1 or higher stage recurrences [103]. In a different study, OTX1 hypermethylation was found in BC cells and linked to poor prognosis (*p* = 0.0451) [104]. ONECUT2 in a 4-gene DNA methylation panel constructed by Wu et al. showed a consistent positive predictive value (PPV) of 100%, negative predictive value (NPV) of 98%, AUC of 0.871, with an SN/SP of 90.5%/73.2% [105]. A different 5-gene DNA methylation panel showed significant results in the detection and prediction of BC in hematuria patients [106]. A 7-gene panel containing also TWIST1 and VIM showed similar results with AUC 0.894, while multivariate analysis proved statistically significant only for HOXA9, ONECUT2, and PCDH17 (*p* = 0.004, 0.004, 0.02, respectively) [107].

Reinert et al. analyzed the methylation status of six genes of BC patients vs. healthy individuals with initial SN/SP of 82–89%/94–100% [108]. However, when recurrence is taken into consideration SN fell to 88–94% and SP to 43–67%. The methylation status of the VIM promoter was also studied by Costa et al., where a combination with GDF15 and TMEFF proved significantly effective to detect BC in tissues and urine, AUC 0.975 [109]. Guo et al. used a 6-gene DNA methylation panel in the examination of urine from hematuria patients, which resulted in SN/SP of 89%/74% [110]. A recent study on BC patients in the Moroccan population showed VIM hypermethylation occurred in 67.14% of tumor samples, while hypermethylation in recurrent and progression cases accounted for 83.34% and 80%, respectively [99]. A study combining miR-663a with VIM proved effective in discriminating BC patients from inflammatory cases, with SN/SP of 80%/75% for miR-663a and SN/SP of 87%/86% for VIM [111]. Chen et al. developed a 2 CpG marker comprising cg21472506 (of OTX1) and cg11437784 (of SOX1-OT) panel for BC detection and surveillance that discriminates BC patients with SN/SP of 90%/83.1%, also showing enhanced sensitivity (64.5%) for LG NMIBC [112].

## 4. Non-Coding RNA

ncRNA is a small RNA molecule that does not translate into a protein. The main types of ncRNAs are transfer and ribosomal RNA (tRNA and rRNA), other types include small RNA molecules such as microRNA (miRNA or miR), long non-coding RNA (lncRNA), and circular RNA (circRNA). Many others belong to this group, but for the purpose of this article, only the mentioned three are included.

Identified in 1993, miRNA is a single-stranded ncRNA molecule with a length of approximately 22 nucleotides [113]. miRNA is a type of 21–23 nt small RNA, which can complement mRNA and either silence it or degrade it. Most miRNAs are down-regulated in BC [114]. As presented in the further examples, seen in Table 4., most miRNA gene expressions have been found to initiate and promote tumor progression. miRNAs may serve as potential diagnostic and prognostic indicators [115,116]. In quantitative reverse transcriptase polymerase chain reaction, microarray can be used as prognostic analysis.

miR-143 can suppress cell proliferation and migration as well as promote apoptosis in BC by inhibiting PI3K/Akt and MAPK signaling and also inhibiting the growth cells by targeting COX-2 which is located on chromosome 5q32 [117,118]. High miR-143 expression was associated with poor survival, as miR-129 simultaneously repressed the tumor suppressor SOX4 and GALNT in BC and the tumor suppressor was downregulated in BC [119]. miR-222 decreased the tumor suppressor PTEN, which was considered to enhance angiogenesis, tumor cell proliferation, and activation of metastasis in BC. Higher expression of miR-222 has been demonstrated in BC than in non-cancerous tissues, giving miR-222 an important role in BC [120]. Higher levels of miR-200 might prevent NMIBC recurrence through the silencing of various target genes, this was reported when the downregulation of miR-200 correlated with metastasis regulation in BC [113]. miR-21 is expressed in essentially all cells, it functions through the regulation of maspin and VEGF-C, suggesting a miR-21/maspin/VEGF-C pathway in BC [121]. Compared with LG tumors (non-muscle invasive), HG tumors (muscle invasive) expressed significantly higher miR-21. miR-21 is also highly correlated with the disruption of tumor-suppressing p53 pathways. miR-21 represses the tumor suppressors PTEN deleted on chromosome 10, tropomyosin 1, and PDCD4 [122]. The importance of miR-203 was suggested when it simultaneously suppressed antiapoptotic factors Bcl-w and Survivin. TaG1 lesions expressed the highest levels of miR-203a-3p and miR-205-5p, also a decrease in expression levels of miR-203a and miR205-5p correlated with the degree of invasiveness of BC [123].

A novel class of RNA is a single-stranded circRNA that is stable, with a long half-life, and can be found in urine [116]. Their role is in the regulation of gene expression via microRNA inhibition. These novel biomarkers have their relative expression levels correlated with tumor grade and stage [124]. Best studied circRNAs in association with BC are listed in Table 5. circRNAs play a pivotal role in the tumorigenesis of BC. In 2016, Zhong et al. published a report on circRNA expression in BC. Using a circRNA microarray expressed in BC and found 469 circRNA. We provide in the table some examples of expressed circRNA in BC [125].

The lncRNAs found in urine have been linked to BC, which deserves further investigation to reveal their functional role in tumorigenesis. Most lncRNA roles are as oncogenes to promote metastasis of BC [118]. The lncRNA 19 is a potential biomarker that is upregulated in the presence of BC, pointing to a crucial role in BC growth. Its expression is significantly upregulated in BC tissues compared with adjacent normal control tissues [126]. The lncRNA 19 is the target gene H19 with a sensitivity of 90.5% [127]. Additionally, a potential biomarker is a lncRNA named urothelial-carcinoma-associated 1 (UCA-1). It regulates cell cycle distribution via CREB through PI3-K dependent pathway and increases chemoresistance of BC cells by regulating Wnt signaling [126]. Using a hybridization assay UCA-1 has a sensitivity of 92.1%. The lncRNA Malat1 has a function to promote cancer cell proliferation, invasion, and metastasis. Malat1 is an important mediator of TGF-β-induced EMT with a sensitivity of 95.7% [128].

## 5. Conclusions

The current recommendation for BC diagnosis includes patients’ history, family history, assessment of symptoms (hematuria, irritation, etc.), urine cytology in conjunction with cystoscopy, and imaging [14]. Even though HG and G3 tumors benefit from high sensitivity (84%), urinary cytology is unsuitable for the detection of LG or G1 tumors with an SN of only 16% [129]. Consequently, invasive examination (cystoscopy) is mandatory, in case of suspicion of BC [14]. Since BC is likely to reoccur, scheduled cystoscopies and other diagnostic or treatment procedures can be lifelong, which poses a significant socioeconomic burden for patients and healthcare professionals, placing BC as the most expensive cancer to treat from diagnosis to death [130]. That is why, the researchers put significant effort to identify reliable markers in terms of sensitivity, specificity, reproducibility, and, what is very important simple utilization in everyday practice at an acceptable cost. Thus far, four FDA-approved markers are used (NMP22, BTA, Urovision, and Immunocyt- in detail in other review articles), which are collectively the most studied markers to date, however, plagued by uncertain results and low SN/SP that can be further enhanced only by combining some of these methods [131,132]. Nevertheless, meta-analysis concludes that these markers suffer from a high rate of false positive cases by nature of their assay design, and may yield false-positive results in 12−26% of patients without BC [133]. Furthermore, their limited SN led up to a missed diagnosis in up to 43% of patients with BC [133]. These data limit their use in everyday practice and hamper their utility as a single tool for reliable BC diagnosis.

Recently in cancer research, overexpression of enzymes has been found in various cancer types [134]. In BC, overexpression of nicotinamide N-methyltransferase (NNMT) in urine is a novel biomarker studied in association with progression and aggressiveness [135]. A known predictor of poor prognosis, Aurora Kinase A affects NNMT expression [136]. Research on NNMT is currently focused on inhibitors, as therapeutic agents of many NNMT-related pathways, such as cancer or diabetes [137,138,139].

Due to the heterogeneity of BC and the requirements of precision medicine, panels of biomarkers are studied to overcome the aforementioned limitations. Some of them report impressive SN, SP, and AUC values [140,141]. To our knowledge, this approach holds the greatest promise but requires further studies, and further validation, especially in the setting of concurrent infection, or other common conditions in clinical settings which can yield different results and differentiate cancer patients from patients with different diagnoses. However, despite the significant effort of researchers, urinary cytology in combination with cystoscopy remains the gold standard for BC diagnosis. Current trends for the identification of miRNAs, cfDNAs, and other subcellular structures, single or in combination (panel) gain interest, and might be the right way to identify novel reliable biomarkers for BC diagnosis and management which are necessary for patients and health care professionals.

## Figures and Tables

**Table 1:** Commercially approved markers.

Marker	Approval	Pooled Statistical Significance	Ref.
BTA stat	FDA	56–83% SN/64–86% SP	[46]
BTA TRAK	FDA	62–76% SN/51–98% SP	[46]
NMP22—(ELISA)	FDA and EAU	71% SN/73% SP	[47]
NMP22—(BladderChek Test)	FDA and EAU	56% SN/88% SP	[48]
Cell Search	FDA	35% SN/97% SP	[49]
UroVysion	FDA and EAU	72% SN/83% SP	[50]
uCyt+	FDA	72.5% SN/65.7% SP	[51]
Uromonitor	EAU	73.5% SN/93.2% SP	[52]

**Table 2:** The most promising DNA markers.

Gene	Modification	Cancer-Associated Gene Function	Statistical Significance	Ref.
FGFR3	mutation, gene overexpression	deregulation of the cell cycle,	93% SN, 86% SP, AUC 0.96 (overall)	[57]
PVRL4	gene overexpression	metastasis	*p* < 0.0001	[58]
CCNE1	copy-number variations	recurrence, progression	*p* = 0.04	[59]
CDKN2A	mutation	deregulation of the cell cycle, progression	95.5% SN, 100% SP	[60]
TERT	mutation	telomere maintenance	80.5% SN, 89.8% SP	[61]
PLEKHS1	mutation, gene overexpression	unknown function	84% SN, 96% SP	[62]

**Table 3:** The most promising epigenetic markers.

Gene	Modification	Affected Variable	Statistical Significance	Ref.
PCDH17, POU4F2, PENK	hypermethylation	detection	87% SN/97% SP	[98]
TWIST1 hTERT	hypermethylation	recurrence/ progression	found in 66.67%/80% found in 83.34%/80%	[99]
DMRTA2	hypermethylation	detection and recurrence	82.9% SN/92.5% SP	[101]
OTX1 + FGFR3, TERT	hypermethylation gene mutation	recurrence/ prognosis	SN 57%—LG-NMIBCSN 83%—T1 and higher	[103]
ONECUT2 + HOXA9, PCDH17, POU4F2	hypermethylation	detection/ progression	90.5% SN/73.2% SP	[105]
ONECUT 2 + OSR1, SIM2, OTX1, MEIS1	hypermethylation	detection/ progression	82% SN/82% SP/ 0.84 AUC	[106]
VIM + GDF15, TMEFF2	hypermethylation	detection	tissue SN/SP—100% urine SN/SP—94%/100%	[109]
VIM + CDH1, SALL3, THBS1, TMEFF2, GDF15	hypermethylation	detection	89% SN/74% SP	[110]
VIM miR-663a	hypermethylation	BC discrimination	87% SN/86% SP 80% SN/75% SP	[111]
cg21472506 and cg11437784	methylation level	BC discrimination surveillance	90% SN/83.1% SP	[112]

**Table 4:** The most promising miRNA markers.

Micro RNA	Role	Expression	Target Gene	Phenotype
miR-143	Tumor suppressor	downregulated	Ras	All grades
miR-129	Proto-oncogene	downregulated	SOX4	High grade
miR-222	Proto-oncogene	upregulated	PUMA	-
miR-21	Proto-oncogene	upregulated	VEGF-C	All grades
miR-200	Tumor suppressor	downregulated	EMT	invasive
miR-205-5p	-	downregulated	PTEN, VEGF-A	All grades
miR-203	Tumor suppressor	downregulated	Bcl-w	All grades

**Table 5:** The most promising circRNA markers.

circRNA	miRNA	Function	Target Gene	Expression
ITCH	miR-17, miR-224	apoptosis	P21, PTEN	downregulated
CIRC0068307	MIR147	stemness	C-Myc	upregulated
NR3C1	miR-27a-3p	Inhibit proliferation	cyclinD1	downregulated
BCRC-3	miR-182-5p	Inhibit invasive	P27	downregulated
Circ0058063	miR-145-5p	proliferation	CDK6	upregulated

## Data Availability

Not applicable.
